# Longitudinal analysis of the Five Sisters hot springs in Yellowstone National Park reveals a dynamic thermoalkaline environment

**DOI:** 10.1038/s41598-022-22047-w

**Published:** 2022-11-04

**Authors:** Jesse T. Peach, Rebecca C. Mueller, Dana J. Skorupa, Margaux M. Mesle, Sutton Kanta, Eric Boltinghouse, Bailey Sharon, Valerie Copié, Brian Bothner, Brent M. Peyton

**Affiliations:** 1 Department of Chemistry and Biochemistry, Montana State University, Bozeman, MT 59717 USA; 2 Thermal Biology Institute, Montana State University, Bozeman, MT 59717 USA; 3 Chemical and Biological Engineering Department, Center for Biofilm Engineering, Montana State University, Bozeman, MT 59717 USA; 4 Department of Biological and Chemical Engineering, Montana State University, Bozeman, MT 59717 USA

**Keywords:** Geochemistry, Biochemistry, Metabolomics, Element cycles, Microbial ecology

## Abstract

Research focused on microbial populations of thermoalkaline springs has been driven in a large part by the lure of discovering functional enzymes with industrial applications in high-pH and high temperature environments. While several studies have focused on understanding the fundamental ecology of these springs, the small molecule profiles of thermoalkaline springs have largely been overlooked. To better understand how geochemistry, small molecule composition, and microbial communities are connected, we conducted a three-year study of the Five Sisters (FS) springs that included high-resolution geochemical measurements, 16S rRNA sequencing of the bacterial and archaeal community, and mass spectrometry-based metabolite and extracellular small molecule characterization. Integration of the four datasets facilitated a comprehensive analysis of the interwoven thermoalkaline spring system. Over the course of the study, the microbial population responded to changing environmental conditions, with archaeal populations decreasing in both relative abundance and diversity compared to bacterial populations. Decreases in the relative abundance of Archaea were associated with environmental changes that included decreased availability of specific nitrogen- and sulfur-containing extracellular small molecules and fluctuations in metabolic pathways associated with nitrogen cycling. This multi-factorial analysis demonstrates that the microbial community composition is more closely correlated with pools of extracellular small molecules than with the geochemistry of the thermal springs. This is a novel finding and suggests that a previously overlooked component of thermal springs may have a significant impact on microbial community composition.

## Introduction

Thermoalkaline springs are unique environments of biological and industrial significance. Commercial applications are well documented in these systems and current thermoalkaline bioprospecting efforts are broad^1^. A successful example is the commercialization of a suite of thermostable enzymes including lipolytic and hydrolytic enzymes^2,3^. Of particular interest is the development of thermostable cellulolytic enzymes capable of converting lignocellulosic biomass into sugars and ultimately ethanol under industrial conditions^4^. The potential to develop thermo- and pH-stable enzymes for commercial applications and interest in the ecology of these systems has led to an accumulation of geochemical and microbial phylogenetic data^5–8^.

Along with bioprospecting efforts, thermoalkaline ecology has also driven investigation. Through this work, temperature has been shown to be a large driver of microbial diversity with increasing spring temperatures translating into a decrease in microbial diversity^9–11^. Temperature increases have also been associated with increases in archaeal abundance and diversity^11,12^. Thermophiles have been shown to tolerate a wide range of pH^10,13^. pH has also been demonstrated to be an important factor in abundance and diversity in thermal environments, with circumneutral and alkaline springs supporting more diverse microbial populations^13,14^. However, these two factors alone do not completely explain the assembly of microbial populations in thermal systems^15^. Thermoalkaline springs have been shown to contain a wide range of bacteria and archaea, with several common clades residing in springs with variable geochemistry and geographical location^9,16^. Predominant microbes in described springs include *Chloroflexi, Deinococcus, Nitrospiral, Cyanobacteria, Proteobacteria, Thermodesulfobacteria, Aquificae, Thermotague, Thermococcales* and *Crenarchaeota*^9,14,16,17^.

Despite increased interest and investigation in these systems, gaps in knowledge remain^2^. For example, the temporal dynamics of thermoalkaline microbial populations across multiple years have rarely been investigated^17–19^ and to our knowledge, analyses combining the microbial with the intracellular metabolome and extracellular small molecule composition have not been conducted. This is especially true of winter sampling of hot springs in YNP where access is limited. Challenges associated with increasing knowledge in thermoalkaline springs include microbial culturing. To confirm the specific metabolic potential and ecological contributions of individual microbes, cultured isolates are often required. Extreme environments like thermoalkaline springs have presented challenges to isolation efforts when using traditional culturing practices, especially with respect to Archaea^16,20^. Gaining extracellular and intracellular-based understanding of thermoalkaline environments has broad implications including improving culturing efforts by providing insight into environmental small molecule and metabolic networks.

Thermoalkaline springs can be found in several locations around the world, including Yellowstone National Park (YNP) where they are prevalent^1,21,22^. One such group of springs in YNP includes the Five Sisters (FS) hot springs, located in the White Creek Drainage (WCD). WCD is part of Lower Geyser Basin, the largest thermal basin in YNP. Thermal features in WCD have been previously identified as areas likely to contain distinct and dynamic environments^23,24^. The FS system is host to a variety of metabolic activity with photosynthesis occurring at the edges of the cooler pools in the spring and summer and heterotrophic activity driven by lignocellulose degradation^6^. In this study, the FS thermoalkaline springs were examined over the course of three years and an extensive analysis was conducted using high-resolution geochemical measurements, 16S rRNA microbial community sequencing, and liquid chromatography mass spectrometry (LCMS) based small molecule characterization that enabled the establishment of temporal microbial trends and a better understanding of driving factors underlying shifts in microbial population makeup and metabolism in this unique environment.

## Results

### Temporal 16S rRNA profiles

**Figure 1:**
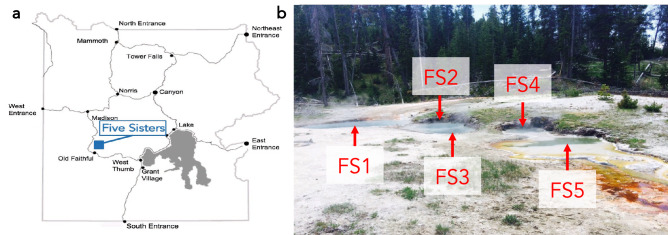
Sampling occurred at the Five Sisters spring system in the White Creek Drainage in YNP. (**a**) Map of YNP indicating the location of the Five Sisters springs north of Old Faithful generated using Adobe Illustrator vCC, adobe.com . (**b**) Picture of the Five Sisters springs with all five pools labeled.

**Figure 2:**
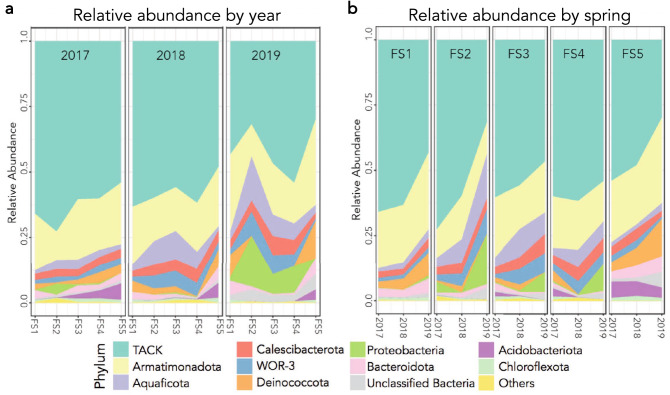
Relative microbial abundances were determined for each spring from 2017 to 2019. (**a**) Phylum-level relative abundance of each spring on a yearly basis. Microbial population composition changes over the three-year period. Each panel represents a year from 2017 to 2019 with springs FS1-FS5 relative abundance levels. (**b**) Phylum-level abundance across years for each of the Five Sisters springs.

**Figure 3:**
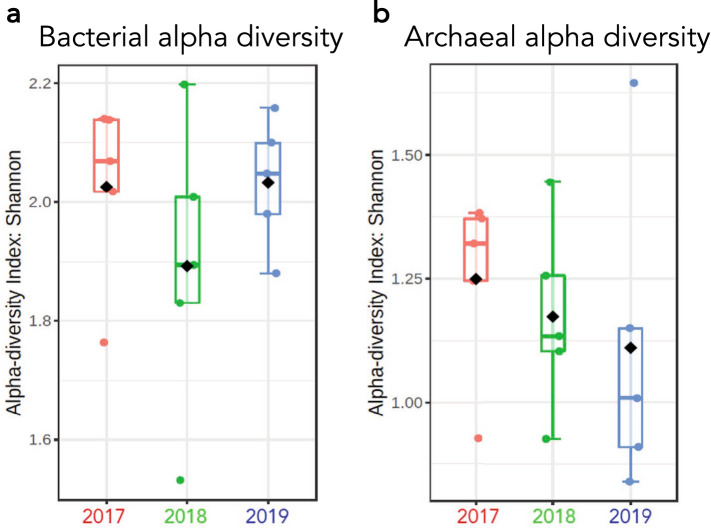
Microbial alpha diversity was calculated using a Shannon Index for all springs each year. (**a**) Bacterial alpha diversity from 2017 to 2019. (**b**) Archaeal alpha diversity from 2017 to 2019. Shannon Index analysis accounts for both the evenness and richness in a system.

To fully document the ecology of thermoalkaline springs, we conducted an extensive analysis of the FS spring system located in Yellowstone National Park (Fig. 1a). This unique site consists of five thermoalkaline pools labeled 1 to 5 from East to West, with variable interconnectivity (Fig. 1b). Samples were collected at the same time of year for three years and geochemical, 16S rRNA and small molecule data were collected. We began our analysis by investigating the microbial community, focusing on populations that showed temporal variation. This revealed several microbial patterns including two striking trends in relative abundance (Fig. 2a). First, a consistent decrease was observed in the relative abundance of microbial ZOTUs from superphylum TACK from 2017 to 2018 and from 2018 to 2019. The second was an increase in relative abundance of ZOTUs from the bacterial phyla Proteobacteria and Deinococcota*.* Over the course of the study, FS1 and FS5 had a marked increase in Deinococcota relative abundance while FS2, FS3 and FS4 had relative Proteobacteria population increases in 2019 (Fig. 2b). Other trends, such as a relative decrease in Acidobacteriota populations in FS3, FS4 and FS5 from 2017 to 2019, were also apparent. Along with a pronounced decrease in abundance, archaeal alpha diversity decreased for each spring between 2017 and 2019 using a Shannon’s index (Fig. 3). The exception to this pattern was FS5, which displayed an increase in alpha diversity in 2019. Although slightly low, at ~ 1.2 for the FS springs, the archaeal alpha diversity was similar to previous studies^11,25^. The 16S rRNA analysis indicated a diverse and elastic microbial community, along with a steady decline in the archaeal diversity from 2017 to 2019.

### Temporal geochemistry of the FS site

**Figure 4:**
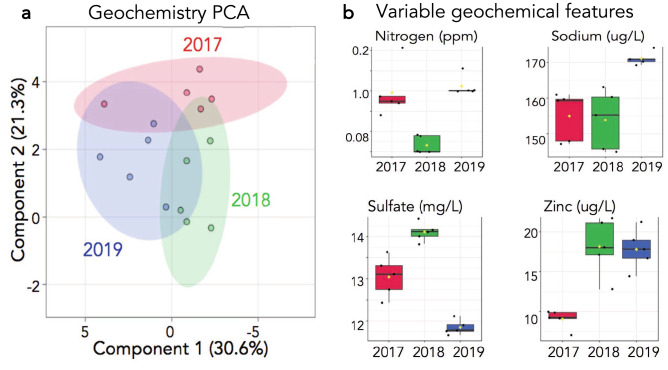
A geochemical analysis was completed at each sampling event. (**a**) 2D-PCA scores plot of all five springs by year. Shaded regions indicate 95% confidence intervals. (**b**) One-way parametric ANOVA analysis indicating geochemical features whose levels are significantly different (p-value < 0.05) between the three years.

The geochemical and small molecule datasets were then examined to determine possible drivers of the temporal and linear archaeal population decreases. We first explored the geochemical data and an initial analysis demonstrated how consistent each spring was over the course of the study (Table 1). Temperature and pH were not significantly different between years and other commonly collected geochemical variables like total carbon, showed little change. Analysis via a 2-dimensional principal component analysis (2D-PCA) scores plot, highlighted the trend of similar geochemistry in 2017, 2018 and 2019 (Fig. 4a). Annual geochemical fluctuations appeared to be moderate although four geochemical measurements including nitrogen, sodium, sulfate and zinc concentrations, were significantly different between the three years using an ANOVA analysis (p < 0.05) (Fig. 4b). However, the patterns of concentration changes between 2017 and 2019 for these variables was not linear. Total nitrogen concentrations decreased in 2018 and then increased back to 2017 levels in 2019 (Fig. 4b). Sodium and zinc both increased in 2019 relative to 2017 but remained constant between 2017 and 2018 for sodium and 2018 and 2019 for zinc. Sulfate concentrations indicated another separate pattern, increasing in 2018 and then decreasing in 2019 to below 2017 levels. Although not included in the PCA or ANOVA analysis, Table 1 also indicates that snowpack, which ultimately turns into spring runoff, was variable between years. Snow water equivalents (SWE) in 2016 were slightly lower than average while 2017 and 2018 were higher than normal with 2018 having higher SWE than 2017. The amount of SWE dictates the quantity of runoff in the following year, i.e., 2016 SWE would impact spring runoff and samples taken in the winter of 2017. The geochemical analysis of the thermal environment indicated a fairly homogenous system over the course of the three years, with no clear pattern emerging except for a steady linear increase in annual snowpack from 2016 to 2018.

**Table 1:** Geochemical characteristics from 2017 to 2019.

Year	February 27, 2017					March 1, 2018					March 7, 2019				
Site	FS1	FS2	FS3	FS4	FS5	FS1	FS2	FS3	FS4	FS5	FS1	FS2	FS3	FS4	FS5
SWE	88%					110%					124%				
Temp (C)	78	78	75	77	66	71	74	73	71	64	75.1	80.3	79.8	80	70
pH	9	8	8	8	8	9	8.5	8.5	8.5	8.5	8.8	8.4	8.4	8.4	8.5
DO (mg/L)	1.7	1.3	1.2	1.3	1.5	0.2	0.2	0.7	0.2	0.8	2.2	0.4	0.5	0.5	4.4
Chloride (mg/L)	216	224.6	234.3	243.1	246.9	253.4	256.2	263.4	255.1	256.5	277.1	296.1	277.2	272	291.1
Sulfate (mg/L)	12.4	12.8	13.3	13.6	14	14.3	14.6	14.7	14.8	15.2	11.7	11.3	11.5	11.4	12
Sodium (mg/L)	158.8	143.3	144.2	157.4	157	140.8	152.4	141.7	161.2	158.1	169.3	170.1	173.6	170	168.2
Potassium (mg/L)	6.9	6.6	6.9	6.7	6.7	6.4	6.8	6.2	7.1	7.2	6.3	6	7.2	7.2	7.4
Zinc (ug/L)	9.5	12.1	11.4	11.5	12	22.4	18.4	19.2	21.9	14.6	19	22	20	16	18
Arsenic (ug/L)	750	670	677	731	715	698	740	667	771	771	800	790	770	780	770
Molybdenum (ug/L)	11.7	11.8	12.3	11.8	11.8	11.7	12.4	11.2	13	13	12	13	12	12	13
TC (mg/L)	45.3	55.3	55.2	55.3	55.7	44.7	54.5	54.1	54.8	54.4	45.7	57	56.6	56	55.4
TN (ppm)	0.2	0.08	0.08	0.09	0.09	0.05	0.06	0.06	0.05	0.05	0.13	0.1	0.1	0.1	0.1
Inorganic Carbon (mg/L)	43.9	54.4	55.2	54.4	55.5	45.3	55	52.4	52.9	54.9	44.1	53.6	53.7	54.3	53.7
NPOC (mg/L)	1.1	0.3	0.2	0.4	0.3	0.4	0.5	0.3	0.5	0.3	0.8	0.6	0.6	0.6	0.5
NH4 (ug N/L)	25	23.1	21.5	23.6	27.8	14	29	20	24	17	32	26	26	24	17

SWE denotes snow water equivalent percentage of the median for the previous year. *DO* dissolved oxygen. *TC* total carbon. *TN* total nitrogen. *NPOC* non-purgeable organic carbon.

### Temporal small molecule profiles

**Figure 5:**
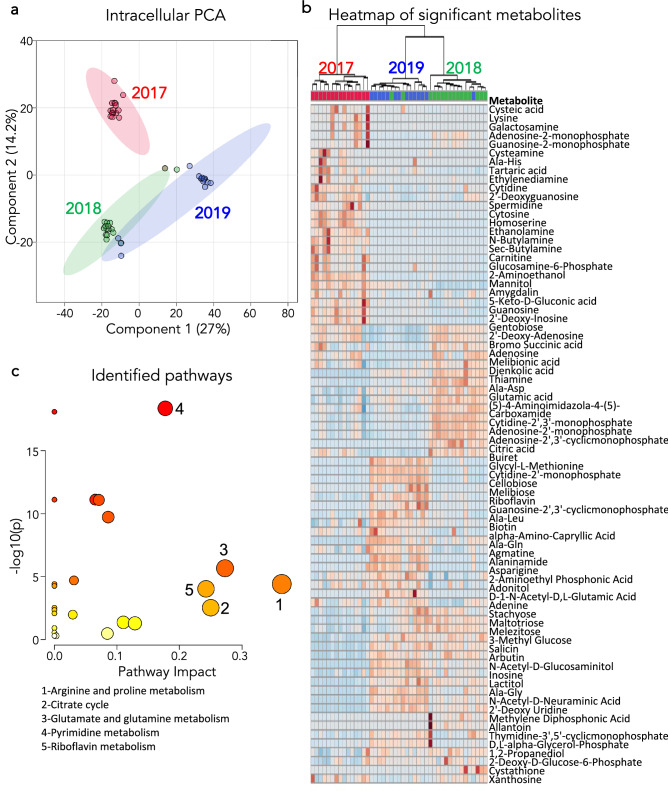
Intracellular small molecules were isolated and metabolomic profiles were analyzed. (**a**) PCA of the intracellular small molecules. Metabolites from 2017 group separately from 2018 and 2019. Metabolites from 2018 and 2019 group independently but overlap in the 95% confidence intervals for several springs, most strongly in FS4 and FS5. (**b**) Heatmap of the significant identified metabolites between years determined using an ANOVA (p < 0.05). Sampling year is indicated at the top of the figure with each column representing one sample and each row representing an identified metabolite. The dendrogram at the top groups 2018 and 2019 separately from 2017 based on the relative abundance of the identified metabolites. The figure was generated using MetaboAnalyst v4.0 (**c**) Metabolic pathways derived from the identified metabolites with the y-axis showing the p-value from an ANOVA of the groups and the x-axis showing the metabolic impact based on the metabolites identified and their role in the specific metabolic pathway.

As the geochemical results did not yield likely factors explaining the archaeal decrease in relative abundance seen in the 16S rRNA data, our next step was to characterize microbial intracellular small molecules using LC–MS. An analysis of intracellular small molecules extracted from cell pellets suggested that a metabolic transition had occurred. A 2D-PCA score plot of the global small molecule metabolic profiles for each year indicated a contrast in metabolic activity between 2017 and both 2018 and 2019 (Fig. 5a). Intracellular small molecules from 2018 and 2019 showed some differences but were more closely related than those from 2017. A functional examination of the metabolomic profiles began with identification of metabolites using a combination of authentic standards and MSMS techniques. An ANOVA analysis was then used to screen the identified metabolites leading to a list of 77 specific metabolites that based on abundance, could discriminate 2017, 2018 and 2019 (p < 0.05) (Supplemental Table 1). To further explore the identified metabolites, a heatmap was generated which indicated similar profiles between 2018 and 2019 relative to 2017 (Fig. 5b). Finally, a pathway analysis using the significant metabolites and their relative concentrations was performed. This yielded 16 pathways with a Holms corrected p-value < 0.05 (Table 2). The impact of the identified metabolites in each pathway was also determined using MetPA (Fig. 5c). This objective analysis of the highlighted pathways revealed 5 significant (p < 0.05) and impactful (impact score > 0.1) pathways: pyrimidine metabolism, glutamate and glutamine metabolism, arginine and proline metabolism, riboflavin metabolism and the citrate cycle (TCA).

**Table 2:** Metabolic pathway analysis.

Pathway	p-value	Impact
Pyrimidine metabolism	1E−17	0.18
Glycerophospholipid metabolism	1.9E−17	0
Glycine, serine and threonine metabolism	1.8E−10	0.07
Lysine biosynthesis	1.8E−10	0
Sulfur metabolism	1.8E−10	0
Purine metabolism	1.8E−10	0.07
Cysteine and methionine metabolism	3.6E−09	0.09
Glutamine and glutamate metabolism	3.9E−05	0.27
Galactose metabolism	3.5E−04	0.03
Arginine and proline metabolism	6.2E−04	0.36
Glutathione metabolism	6.2E−04	0
Thiamine metabolism	7.7E−04	0
Riboflavin metabolism	1.2E−03	0.24
Citrate cycle	3.5E−02	0.25
Glyoxylate and dicarboxylate metabolism	3.5E−02	0
Cyanoamino acid metabolism	4.4E−02	0

**Figure 6:**
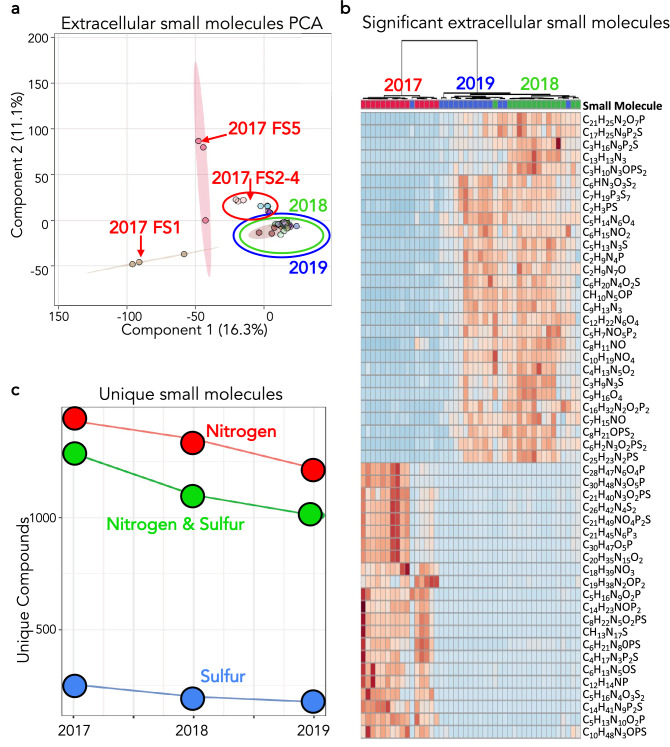
Extracellular small molecules were isolated and small molecule profiles were analyzed. (**a**) PCA for extracellular small molecules in the sediment. 2018 and 2019 group very closely together relative to 2017. FS2-4 from 2017 group together and are closer to 2018 and 2019 than FS1 and FS5 from 2017. (**b**) Heatmap of the top 50 discriminating small molecules between 2017, 2018 and 2019. The top of the figure showing the year of each sample with a dendrogram indicating that 2017 groups independently from 2018 and 2019. The figure was generated using MetaboAnalyst v4.0 (**c**) Plot showing unique sulfur, nitrogen and sulfur and nitrogen containing molecules. Element specific molecules are shown in each year from 2017 to 2019. Formulas were determined from the mass spectrometry datasets using an error of 15 ppm.

Extracellular small molecules extracted from the sediment exhibited a similar trend wherein 2017 was markedly different than 2018 and 2019 (Fig. 6a). The variation between years was most pronounced in the 2017 FS1 and FS5 sediment environments. Due to the unique molecular makeup of the extracellular small molecule profile, chemical formulas rather than identifications were determined. Exploring chemical formulas for the top 50 small molecules that best differentiate years via a heatmap indicated abundance changes in specific elemental nitrogen- and sulfur-containing species in spring sediment from 2017 relative to 2018 and 2019 (Fig. 6b). Of the top 50 discriminating small molecules based on an ANOVA, only two did not contain either nitrogen or sulfur. This led to an investigation into nitrogen- and sulfur-containing species in the small molecule profiles and a general trend indicating a decrease in the number of unique nitrogen-, sulfur-, and combined nitrogen and sulfur-containing compounds in the thermal sediments each year of the study (Fig. 6c). Nitrogen-containing compounds exhibited a 15% decrease between 2017 and 2019, while sulfur-containing compounds exhibited a 30% decrease over the same time period. The mass spectrometry-generated small molecules data held a wealth of information, from which emerged a consistent trend where the number of unique extracellular nitrogen- and sulfur-containing small molecules decreased each year in the FS system.

### Correlation analysis

Correlograms were built to establish whether statistically important variables within the 16S rRNA data correlated with variables from the three corresponding temporal datasets. Correlograms were used to provide a rationale as to the possible mechanisms of archaeal decline in the FS springs over time. The top 10 most discriminating variables between year and springs were selected from the geochemistry dataset and correlated with 16S rRNA sequencing information (Supplemental Fig. 1). This figure was created using ZOTUs but is shown by phylogenetic order. Seven of 10 bacterial and archaeal ZOTUs exhibited a significant positive correlation with sulfate concentrations (Supplemental Fig. 1). Zinc concentrations were similarly associated with an increase in the top 10 microbial variables, though this trend was not as pronounced as with sulfate. Sodium, arsenic and dissolved oxygen exhibited an opposite trend, with a negative correlation with most of the selected ZOTUs.

**Figure 7:**
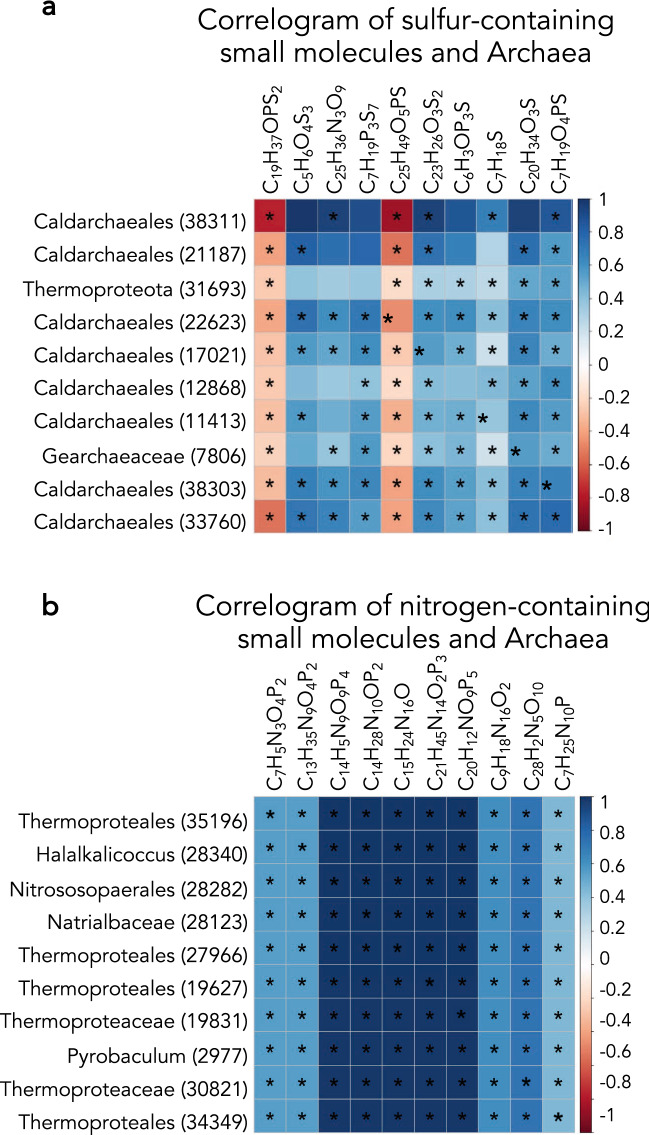
Data from all available datasets were examined to determine correlative features. (**a**) Correlogram as described in the methods section investigating archaeal ZOTUs and sulfur containing extracellular small molecules. (**b**) Correlogram of archaeal ZOTUs and nitrogen containing extracellular small molecules. The intensity of the color indicates the strength of the correlation, with blue demonstrating positive and red demonstrating negative correlations. An asterisk indicates a p-value of < 0.05 for the correlation. Correlograms were created using the mixOmics package in R.

**Figure 8:**
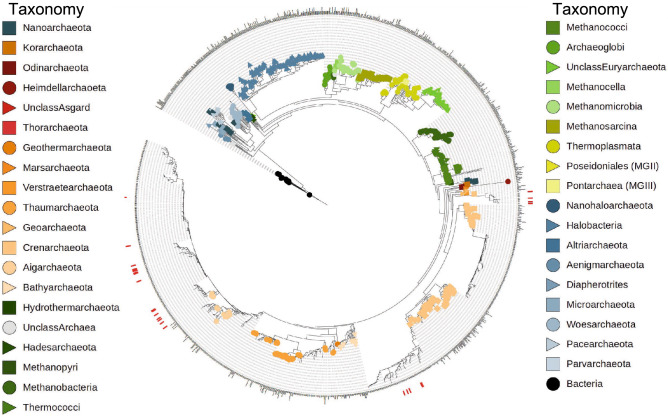
A phylogenetic tree of archaeal sequences was generated and ZOTUs that correlated strongly to the extracellular small molecule datasets were identified in the tree. The Inner ring is populated by Archaea found in the Five Sisters site using metagenomic data. The colored shapes in the inner ring indicate phyla of previously cultured and characterized archaeal species corresponding to the taxonomic classifications shown. Archaeal sequences isolated in the correlogram analysis are indicated by red dashes in the outer ring. The figure was created using Interactive Tree of Life v6.

A further correlative analysis of the small molecule datasets revealed strong relationships between specific nitrogen- and sulfur-containing compounds and Archaea, the majority of which were overwhelmingly positive (Fig. 7). Not only were almost all correlations positive, i.e., decreases in specific small molecules were observed with decreases in archaeal relative abundance, but the majority of the correlations were significant with a p-value < 0.05. An archaeal phylogenetic tree with the top 25 discriminating ZOTUs between years that had positive correlations to annual concentrations of nitrogen- and/or sulfur-containing compounds indicated that the ZOTUs with significant correlations belonged to two separate clades of Archaea the phyla *Aigarchaeota* and *Crenarchaeota*, both in the TACK superphylum (Fig. 8)^22^. This analysis indicated a relationship between specific nitrogen- and sulfur-containing extracellular small molecules and distinct clades of *Aigarchaeota* and *Crenarchaeota*.

## Discussion

This study was undertaken to explore thermoalkaline hot springs and the microbial life that inhabits them to better understand the ecology and small molecule biology of these unique environments. The described analysis allowed for the most comprehensive view to date of microbial life in the FS spring system. Our data indicate that a systemic change likely occurred between 2017 and 2018 which significantly impacted the environment and microbial ecology of the FS springs. Samples from 2017 to 2018 displayed coordinated extracellular small molecule, microbial and intracellular small molecule shifts. Yet, these general trends did not fully explain the pattern of archaeal decline relative to that of bacterial populations. This phenomenon of differential responses between bacteria and archaea to a shared environmental stimulus has been observed by Pala, et al., where contrasting archaeal and bacterial abundance shifts were the result of differing geochemical factors in the same aqueous environment^26^.

However, the geochemical variables with significant differences over the course of the study did not exhibit a characteristic pattern reflective of the archaeal population data. To discover a correlative pattern for archaeal decline, we then examined the intracellular and extracellular small molecule profiles. An initial examination revealed a global shift between 2017 and 2018, suggesting environmental and metabolic change(s) in the microbial communities of the spring system during this time period. A closer investigation into the intracellular data revealed disruption in several specific nitrogen and sulfur cycle pathways. Pathways found to be impactful and significant included pyrimidine metabolism, glutamate and glutamine metabolism, arginine and proline metabolism, riboflavin metabolism and the citrate cycle. These pathways all have connections to both energy metabolism and nitrogen cycling as well as other metabolic impacts^27,28^. Disruption in the abundance of metabolites in these pathways could be due to or could cause shifts in archaeal abundance, especially with pathways impacting nitrogen cycling, where Archaea have been shown to play key roles^29^. Specifically, Archaea have unique enzymes and pathways in riboflavin synthesis as well as in nitrogen assimilation and dissimilatory pathways which could modulate the amino acid metabolism as well as pyrimidine metabolism and the citrate cycle^30,31^. Although not classified as impactful in our MetPA, sulfate metabolism was also found to be significantly (p < 0.05) dysregulated between years and is known to have different enzymes and pathways between bacterial and archaea metabolism^32^.

While the initial analysis of intracellular and extracellular small molecules demonstrated a shift in metabolism and the spring environment between 2017 and 2018, it did not fully explain the consistent loss of Archaea from 2017 to 2019. Our analysis shows that the strongest correlations to archaeal decline are to specific nitrogen- and sulfur-containing small molecules. These small molecules decreased steadily from 2017 to 2019 and coincided with a general decrease in the relative abundance and diversity of Archaea. This observation led to the hypothesis that environmental shifts may induce disparate small molecule profiles with contrasting elemental composition and structure, which could result in thermophiles in the FS system adapting metabolic strategies to environmental changes. Availability of bioactive small molecules would then lead to differential success of specific organisms in the FS system, which could explain the shift in population makeup that occurred from 2017 to 2019 as defined by the observation of a decline in the relative abundance of Archaea.

Although a general decline in Archaea was noted, two clades of Archaea exhibited a strong positive correlation to the number of nitrogen and sulfur compounds detected. These Archaea belonged to either *Aigarchaeota* or *Crenarchaeota*, both members of the TACK superphylum^33^. The top correlating Archaea did not include any members from the other superphyla DPANN, *Euryarchaeota* or *Asgard*^34^. Many members of the TACK clade have shown strong functional roles in nitrogen and sulfur cycles, such as ammonia oxidation (*Thaumarchaeota*), sulfur oxidation (*Crenarchaeota*), dissimilar sulfur metabolism (*Korarchaeota*) and facultative nitrate reduction (*Geoarchaeota*)^1,29,35,36^. The correlations to nitrogen- and sulfur-containing compounds coupled with previously discovered metabolic traits of the TACK superphylum demonstrate the need to further explore the relationship that may exist among the species of *Aigarchaeota* and *Crenarchaeota* in the FS system with respect to nitrogen and sulfur metabolism. In particular, the globally distributed but as of yet uncultivated *Aigarchaeota*^37^ have consistently been found in high relative abundances within thermoalkaline sites within Yellowstone^17,38^. Based on metagenomics and single cell assemblies, this group has high metabolic versatility, including pathways autotrophy, dissimilarity sulfite reduction and carbon monoxide oxidation^39^, as well as diversity in carbon substrate utilization, including acetate, fatty acids and amino acids^40^, which could play a large role in nutrient cycling in these systems.

While the decline in nitrogen- and sulfur-containing extracellular small molecules could be due to abiotic or biotic influences, Gonisor et al. concluded that the chemodiversity seen in nearby Octopus Spring was likely not microbially driven^41^. Their interpretation was based on the low biodiversity of thermoalkaline springs and the high level of unique extracellular small molecules. One potential explanation for the loss of nitrogen and sulfur compounds is the differential annual mixing of ground and surface water in the springs, where runoff, groundwater and snowpack can impact spring geochemistry and extracellular small molecule composition^41,42^. In our study, samples were collected in late February and early March. Our sampling coincides with very low surface runoff which occurs in late springs at this site. The average ambient temperature during our sampling times was still well below freezing so no appreciable addition melting should have occurred such that the spring system environment should be stable at the time of collection. However, the amount of groundwater recharge from the previous year may have a significant impact on resulting spring water composition the following winter. The area around WCD had unusually high snowpack in 2017 and 2018 (impacting 2018 and 2019 data) relative to the previous and following years according to the Water Resources Data System & State Climate Office of Wyoming (www.wrds.uwyo.edu). On March 1st, 2017 and 2018, the snow water equivalent amounted to 110% and 124% of the average for the time of year in, respectively. Snow water equivalent in 2016 was 88% of the median, potentially impacting 2017 data. This SWE trend indicating an increased snowpack from 2016 to 2018 correlates with the loss of unique nitrogen- and sulfur-containing compounds and archaeal decline. A Pearson correlation analysis found a strong negative correlation of − 0.99 between SWE and archaeal alpha diversity with a p-value of 0.0031. During the increased runoffs in the 2017 and 2018 season from high snowpack, the hot spring water composition likely went through a significant change leading to distinct differences in environmental small molecule compositions. Runoff from snowpack has been previously theorized to modulate specific populations of Archaea in hot springs. Campbell et al*.* observed temporal changes in *Sulfolobus islandicus* populations within different hot springs in YNP that did not correlate with measured geochemical changes observed in the springs^43^. These changes were hypothesized to arise from runoff or other hydrogeochemical perturbations.

In conclusion, our analysis has characterized some of the complexity and dynamism of thermoalkaline spring ecology. It also revealed that thermophilic Archaea may be sensitive to small environmental perturbations. The combination of standard geochemical techniques with novel mass spectrometry small molecule analysis exposed the limitations of solely using elemental composition and standard geochemical measurements, such as dissolved oxygen, to assess changes in complex environmental systems. Mass spectrometry analysis identified specific changes in small molecule composition that correlated with archaeal relative abundance. Such associations would have been missed using a purely geochemical analysis. Our comprehensive study revealed that environmental events, possibly related to snowpack, occurred between 2017 and 2019 that shifted hot spring compound composition, manifesting in nitrogen- and sulfur-containing compound transitions, correlating with metabolic changes and a relative decrease in Archaea.

## Methods

### Description of the Five Sisters Site

The FS site (Yellowstone Research Coordination Network Database LWCG023A, LWCG023B, LWCG023C) consists of a group of alkaline-chloride springs located in the south-east corner of the Lower Geyser Basin in the WCD (44.5325°N, 110.7971°W. White Creek flows down the drainage and is accompanied by several thermoalkaline springs including Spindle Geyser (LWCG149) and two of the best studied sites in YNP, Octopus Spring (LWCG138) and Mushroom Spring^44^. The FS site is located a few meters south of White Creek against a steeply inclined hill and consists of three springs and five distinct pools (FS1-FS5). FS1 is fed by a small geyser and is connected above-ground to FS2 and FS3. There is no visible above-ground connection between FS3 and FS4, although there may be below-ground connectivity between the two springs. FS4 and FS5 are connected above-ground and FS5 outflow continues away from the spring group and feeds into White Creek. FS1 is the largest spring at several meters wide and deep, followed by FS5, FS3, FS2 and FS4.

### Sample collection

Samples were collected within one week of the first day of March in 2017, 2018 and 2019. By sampling at the same time of year, this analysis allowed for a long-term look at non-seasonal changes in a thermoalkaline spring. Stainless steel cups on extendable poles were used to collect samples from each spring. The cups were sterilized with 70% ethanol before each sampling trip, rinsed with spring water first and then used to collect a small amount of sediment slurry. Samples were collected in triplicate and were taken at three distantly located locations within each spring with paired subsamples for metabarcoding and mass spec analysis collected at each sampling location. The same sampling sites within each spring were used for all three years in the analysis. 15 mL of sediment slurry was collecting for each sample which was composed of ~ 8 mL of sediment with ~ 7 mL of thermal water. Collected samples were placed in sterile 15 mL conical centrifuge tubes (Corning, Corning, NY). Slurry samples were immediately frozen in a dry ice and ethanol bath in the field and stored on dry ice until transported to Montana State University (MSU) and stored at − 80 °C until genomic or LCMS analysis.

### Geochemical analysis

Aqueous geochemistry was monitored with each sampling event. Briefly, the temperature and pH of each site was measured in situ using a combined pH-temperature probe (Hach HQ30d, Hach Co., Loveland, CO). Dissolved oxygen was measured in the field using the High Range Dissolved Oxygen method and a portable colorimeter (Hach DR900, Hach Co., Loveland, CO) (HELM). Total dissolved metals were analyzed using 0.22 µm filter-sterilized water acidified with 5% trace metal-grade nitric acid. Concentrations of total metals were quantified using an Agilent 7500ce ICP-MS by comparing to certified standards (Agilent Technologies, Environmental Calibration Standard 5183–4688) at MSU’s Environmental and Biofilm Mass Spectrometry Facility. Samples for anion analysis were filtered through 0.22 µm filters, and the filtrate was analyzed using a Dionex ICS-1100 chromatography System (Dionex Corp., Sunnyvale, CA) equipped with a 25 μL injection loop and an AS22-4 × 250 mm anion exchange column, using an eluent concentration of 4.5 mmol/L sodium carbonate and 1.4 mmol/L sodium bicarbonate flowing at a rate of 1.2 mL/min. Samples for total carbon (TC), total nitrogen (TN), non-purgeable organic carbon (NPOC), and dissolved inorganic carbon (IC) were filtered through 0.22 µm filters, and the filtrate deposited in ashed glass vials filled with no headspace and capped with a septum. A Shimadzu TOC-CSH instrument with an attached TN module (Shimadzu Scientific Instruments, Columbia, MD) was used to measure TC/TN/NPOC/IC at MSU’s Environmental Analytical Lab (EAL). Filtered samples acidified with sulfuric acid (final pH < 2) were also sent to the EAL for ammonium analysis using a Lachat QuickChem 8500 flow injection analyzer (Hach Co., Loveland, CO).

### DNA extraction and metabarcoding analysis

Each spring was sampled in triplicate every year of the study and each of these samples was used for metabarcoding analysis. DNA was extracted from each replicate using the FastDNA™ Spin Kit for Soil kit (MP Biomedicals) according to the manufacturer’s instructions, with an additional 40 s bead beating step. The V4 region of the 16S rRNA gene was targeted using the latest versions of the 515F-80R primers^45^, 515F-A (GTGYCAGCMGCCGCGGTAA;^46^) and 806R-B (GGACTACNVGGGTWTCTAAT;^47^) using Phusion Hot Start II Hi Fidelity polymerase in 25 ul reactions. These primers are the most widely accepted in the field of microbial ecology^46–48^ and have consistently been found to have both discriminatory power and low bias against specific taxonomic groups^49^. Thermocycling conditions were an initial denaturation at 98 °C for 30 s, 22 cycles of 98 °C denaturation for 15 s, annealing at 58 °C for 30 s, extension at 72 °C for 20 s, with a final extension at 72 °C for 5 min. To facilitate multiplexing, dual-index barcodes were added in a second PCR using the Nextera kit (Illumina Inc.) with 10 cycles as above, but with an annealing temperature of 55 °C. PCR reactions were quantified using the Quant-It HS dsDNA kit (Invitrogen) and measured using a Biotek H2 plate reader. Reactions were pooled at equal concentrations and sequenced on a MiSeq using V3 600 cycle kits.

Paired-end reads were merged, primers were removed, and sequences were quality filtered with an expected error rate of 0.5 using USEARCH version 11^50,51^. Operational taxonomic units (OTUs) were identified using UNOISE version 3, which identifies biological sequences clustered into zero-radius OTUs (ZOTUs), similar to amplicon sequence variants. ZOTU tables were generated by mapping reads back to representative ZOTU sequences. Taxonomy of ZOTUs was identified using the IDTAXA online classifier via DECIPHER version 2.20.0(http://DECIPHER.codes)^52^. Archaeal sequences were added to a reference tree generated using full and near full-length 16S sequences downloaded from GenBank and the Genome Taxonomy database^53^. Sequences were aligned using MAFFT version7.487 and the reference phylogeny was constructed using maximum likelihood with RAxML version 8.0^54,55^. Environmental sequences were aligned to the reference and added to the tree using pplacer, version 1.1 and the combined trees were annotated using the Interactive Tree of Life version 6 (http:www.itol.embl.de)^56,57^. Statistical analysis was accomplished by exploring the 16S rRNA ZOTU relative abundance data using MicrobiomeAnalyst to determine year and spring specific microbial trends^58^.

### Sediment small molecule extraction

Similar to the metabarcoding analysis, three collected samples were used from each spring for each annual analysis. Analysis of all annual samples occurred concurrently. Sediment samples were extracted using several methods resulting in small molecule fractions that were characterized by LCMS analysis. Extraction began by thawing frozen samples in a 40 °C water bath. Microbes were dislodged through two 1-min rounds of agitation on a vortex machine. Additional agitation was not found to increase intercellular small molecule yields. Well-mixed samples were then centrifuged for 5-min at 400 RPM to create a sediment pellet. The clear supernatant was then collected and placed in a clean vial and the procedure was repeated with the addition of a 5 mL Milli-Q water wash. Wash supernatant was added to the original sediment-free supernatant. Combined supernatant was then centrifuged at 10,000 RPM for 15 min to create a cell pellet. The supernatant was collected in a clean vial for extracellular solid-phase extraction (SPE) analysis and enough phosphate buffer solution (1X PBS) was added to the cell pellet to completely cover the pellet.

The extracellular layer was first acidified to a pH of 2 using formic acid (Fisher Chemical, Hampton, ND) and Agilent Bond Elut PPL solid phase extraction cartridges (Agilent Technologies, Santa Clara, CA) were prepared. Preparation involved two cartridge volume additions of methanol (Fisher Chemical, Hampton, NH) followed by two cartridge volume additions of Milli-Q water and a final cartridge volume addition of methanol. SPE cartridges were then connected to a SPE manifold (VacMaster 10, Biotage, Uppsala, Sweden) and a vacuum pump to selectively concentrate and isolate extracellular small molecules. Acidified samples were passed through the cartridges and then placed under N_2_ to dryness. Clean vials were placed below the cartridges and captured extracellular small molecules were eluted using 1 mL of methanol. Extracellular small molecules were further concentrated under negative pressure using a Concentrator Plus (Eppendorf, Hamburg, Germany) until dry and then stored at − 80 °C until ready for LCMS analysis.

The vial containing the cell pellet was centrifuged at 400 RPM and the PBS supernatant was removed. Two cell pellet volumes of extraction buffer were added consisting of 8 M urea (Fisher Chemical, Hampton, NH), 0.1 M Tris–HCL (MilliporeSigma, Munich, Germany), 50 mM ethylenediaminetetraacetic acid (EDTA) (MilliporeSigma, Munich, Germany) and 1X protease inhibitor mix (MilliporeSigma, Munich, Germany). Cell pellets were next lysed in a two-part procedure. First, cell pellets in extraction buffer were placed in liquid nitrogen for 10 s then removed and allowed to thaw. This procedure was repeated three times. After the freeze/thaw procedure, cell pellets underwent two rounds of sonication using a Biologics Inc. Ultrasonic Homogenizer 3000 (Bioloics, Manassas, VA) set at a 40% duty cycle for 3 min.

After cell lysis, the intracellular samples were centrifuged at 15,000 RPM for 30 min to pellet cell debris. The resulting supernatant was removed and placed in a clean vial while the debris were washed with the addition of 50µL of extraction buffer. After agitating the debris with a vortex machine, samples were centrifuged at 15,000 for 30 min. The second supernatant was again removed and added to the vial containing the first supernatant. Four sample volumes of ice-cold acetone (Fisher Chemical, Hampton, NH) were then added to precipitate protein. Samples were placed in a − 80 °C freezer for 2 h to aid precipitate formation. After 2 h, samples were centrifuged at 5000 RPM for 5 min and the intracellular supernatant was collected and placed in a clean vial. As with the extracellular small molecule layer, the intracellular small molecule layer was concentrated under negative pressure to dryness and stored at − 80 °C until LCMS analysis. When ready for LCMS analysis, both the intracellular and extracellular small molecule samples were reconstituted with 50 µL of methanol:water (50:50) and placed in clean MS vials.

### LCMS analysis

Samples were analyzed on an Agilent 6538 Q-TOF MS paired with an Agilent 1290 ultra-high performance liquid chromatography (UHPLC) (Agilent Technologies, Santa Clara, CA) using a 132 Å, 2.2 μm, 2.1 mm × 150 mm Cogent Diamond Hydride HPLC column (Microsolv, Greater Wilmington, NC) located in the Proteomics, Metabolomics and Mass Spectrometry facility at MSU. Ionization was accomplished via electrospray ionization in positive mode. Mobile phases A and B consisted of water with 0.1% formic acid and acetonitrile with 0.1% formic acid, respectively. A 15-min UHPLC run time was used, starting with 100% mobile phase B and moving to 30% B in a linear gradient over 14 min. At 14 min, mobile phase B was increased back to 100% for the final minute of the UHPLC run. Flow was kept at 600 µL/min and the column compartment temperature was constant at 30 °C. Samples were ionized using electrospray ionization with a capillary voltage of 3500 V and a source temperature of 150 °C with nitrogen as a desolvation gas at 350 °C. A range of spectra were collected from 50 to 1000 m/z. Pooled samples were ran throughout the analysis and observed for continuity during the sample analysis. Pooled samples were also used to calculate coefficients of variation from concentrations of a randomly selected group metabolites and a mean value of 4.8% was calculated.

LCMS raw data files were converted to .mzML files using MSConvert version 3.0 with Vendor peak picking and data mining was completed using mzMine version 2.53^59,60^. A minimum intensity of 1,000 counts was used throughout the mining process along with a ppm error of 20 and a time discrepancy of 0.1 min for determining unique peaks. Molecular formulas were determined using mzMine’s formula generation identification feature with an error of 15 ppm. To validate the molecular formula assignments and the presence of nitrogen and sulfur, MS features from 50 small molecules were examined (Supplemental Tables 2 and 3). Each of these small molecule features was selected in the following statistical correlations section and all possible organic molecular formulas were derived using the m/z value and a 15 ppm cutoff^61^. 79% of the possible formulas for the m/z values of the predicted sulfur group contained sulfur. Nitrogen was even more consistent, as 89% of the possible formulas included nitrogen. Although several formulas were possible for many of the m/z mass values measured by LCMS, the presence of nitrogen or sulfur was consistent in the majority of possible formula assignments.

After data mining, blank samples were used to remove residual features from the datasets. Features were only kept in the experimental data if they had an area five times greater than the blank sample. Using this method, almost 5,000 combined features were found in the extracellular small molecule samples and almost 3,200 features were found in the combined intracellular small molecule samples. Metabolite identification was based on accurate mass and retention time matches with authentic standards from an in-house library. The in-house library contained over 400 metabolites analyzed on the same same LC column, instrument, and method as the sample analysis. A 15 ppm error and 0.25 min retention time window were used to positively annotate features. Datasets were then grouped and analyzed using MetaboAnalyst version 4.0 (http:www.metaboanalyst.ca)^62^. Features were removed if not in over 50% of the samples. Data were next filtered using their interquartile range value and normalized by sum and autoscaling. Pathway analysis was also completed in MetaboAnalyst using a Holms p-value (p < 0.05) to determine significance of each pathway. The Holms p-value is a corrected value that accounts for multiple comparisons. A Metabolic Pathway Analysis (MetPA) score (impact score > 0.1) was also calculated to objectively elucidate impactful pathways based on the importance of the identified metabolites in each specific pathway^63,64^.

### Correlation analysis

The geochemical, 16S rRNA, intracellular and extracellular small molecule datasets were compared using correlograms. Correlograms identify variables that discriminate groups, in this case by year, and then calculate the strength and statistical significance of the correlation. This analysis was accomplished by utilizing Bioconductor v3.15 and mixOmics v6.1 packages in R (http:www.bioconductor.org) (http:www.mixOmics.org)^65,66^. Two datasets were compared at a time and the top discriminating features were determined using a partial least-squares discriminating analysis (PLS-DA). The top features from each dataset were then compared between springs and years to determine their correlative relationship. An ANOVA analysis was also conducted for each relationship to determine the significance of the correlation.

## Supplementary Information

Supplementary Information.

## Data Availability

The nucleotide sequence data reported in this study are available in the MG-RAST database under the accession number mgm4929532.3 [www.mgrast.org/mgmain.html?mgpage=project&project=mgp98643]. Small molecule and metabolomics data reported in this study are available in the MetaboLights database under the identifier MTBLS5344 [www.ebi.ac.uk/metabolights/MTBLS5344].
