# Supplementary material for: Longitudinal analysis of the Five Sisters hot springs in Yellowstone National Park reveals a dynamic thermoalkaline environment
Source: Sci Rep. 2022 Nov 4;12:18707. doi: 10.1038/s41598-022-22047-w (PMC9636164; doi:10.1038/s41598-022-22047-w)
Supplement: Supplementary file 1 — Supplementary Information. [file 41598_2022_22047_MOESM1_ESM.pdf]

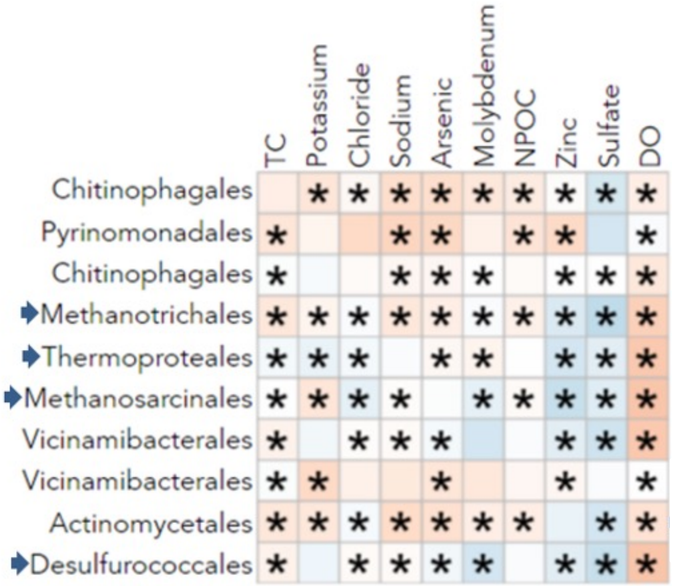

Supplemental Figure 1. Correlogram of the 16S rRNA data and the geochemical data. Blue squares indicate positive correlations while red squares indicate negative correlations. An asterisk is shown if the correlation has a p-value of <0.05. 16S rRNA ZOTUs are shown at the phylogenetic level of order. Archaeal orders are noted with arrows.

Supplemental Table 1. Discriminating ($p<0.05$) metabolites between years

Metabolite	p.value
(5) 4-Aminoimidazola-4(5)-Carboxamide	6.58e-05
1,2-Propanediol	5.55e-03
2-Aminoethanol	1.43e-17
2-Aminoethyl Phosphonic Acid	1.73e-04
2-Deoxy-D-Glucose-6-Phosphate	3.18e-02
2'-Deoxy Adenosine	2.51e-08
2'-Deoxy Inosine	1.42e-07
2'-Deoxy Uridine	2.59e-13
2'-Deoxyguanosine	2.11e-04
3-Methyl Glucose	2.60e-11
5-Keto-D-Gluconic Acid	1.27e-05
Adenine	3.47e-04
Adenosine	3.47e-03
Adenosine-2'-monophosphate	8.80e-03
Adenosine-2',3'-cyclicmonophosphate	3.98e-07
Adonitol	1.77e-02
Agmatine	2.21e-05
Ala-Asp	2.18e-08
Ala-Gln	2.27e-06
Ala-Gly	2.93e-11
Ala-His	2.67e-02
Ala-Leu	2.81e-06
Alaninamide	1.98e-15
Allantoin	3.36e-02
alpha-Amino-Capryllic Acid	2.02e-02
alpha-GlycerolPhosphate	2.64e-06
Amygdalin	1.83e-02
Arbutin	2.89e-10
Arginine	6.17e-04
Asparagine	2.62e-10
Biotin	9.68e-04
Bromo Succinic Acid	5.17e-03
Buiret	2.75e-04
Carnitine	1.96e-05
Cellobiose	1.56e-10
Citric Acid	3.41e-08
Cystathione	6.76e-03
Cysteamine	1.49e-03
Cysteic Acid	6.31e-03
Cytidine	1.62e-06
Cytidine-2'-monophosphate	1.16e-10
Cytidine-2',3'-monophosphate	2.51e-09
Cytosine	1.40e-11
D-1-N-Acetyl-D,L-Glutamic Acid	6.13e-05
Djenkolic Acid	7.60e-15
Ethanolamine	2.08e-09
Ethylenediamine	4.66e-03
Galactosamine	2.08e-03
Gentiobiose	2.69e-08
Glucosamine-6-Phosphate	9.57e-04
Glutamic Acid	3.64e-07
Glycyl-L-Methionine	9.94e-10
Guanosine	1.55e-09
Guanosine-2'-monophosphate	3.55e-02
Guanosine-2',3'-cyclicmonophosphate	4.50e-06
Homoserine	9.11e-11
Inosine	1.55e-09
Lactitol	4.01e-10
Lysine	1.80e-03
Maltotriose	2.41e-07
Mannitol	1.67e-10
Melezitose	3.33e-06
Melibionic Acid	3.35e-02
Melibiose	5.70e-07
Methylene Diphosphonic Acid	8.20e-03
N-Acetyl-D-Glucosaminitol	2.46e-15
N-Acetyl-D-Neuraminic Acid	7.78e-14
N-Butylamine	2.13e-08
Riboflavin	7.11e-09
Salicin	5.62e-12
Sec-Butylamine	4.08e-06
Spermidine	2.17e-04
Stachyose	9.17e-05
Tartaric Acid	7.65e-05
Thiamine	9.84e-12
Thymidine-3',5'-cyclicmonophosphate	8.82e-04
Xanthosine	1.85e-02

Supplemental Table 2. Nitrogen formula determination

m/z	Formula	Formulas Containing N(%)
192.158	C9H21NO3	75
223.987	C5H7NO5P2	83
347.201	C16H32N2O2P2	83
153.120	C5H16N2O3	86
218.138	C10H19NO4	71
301.124	C9H20N2O9	82
317.192	C6H25N10O3P	87
128.070	C4H7N4O	50
164.116	C4H13N5O2	100
231.082	C5H14N2O8	100
164.117	C9H13N3	100
577.412	C20H48N16O4	93
121.063	C2H9N4P	100
130.121	C7H15NO	100
112.050	C4H5N3O	100
209.092	C10H12N2O3	83
301.145	C11H22N6P2	93
372.219	C8H21N17O	90
277.103	C5H13N10O2P	94
532.337	C23H46N7O5P	92
312.206	C13H26N7P	87
782.483	C24H64N17O10P	95
284.172	C12H21N5O3	80
482.260	C16H36N9O6P	93
149.037	C4H8N2O2S	100
	Average	89

Supplemental Table 3. Sulfur formula determination

m/z	Formula	Formulas Containing S(%)
415.140	C23H26O3S2	81
493.311	C25H49O5PS	85
90.976	C2H3PS	50
216.919	C6H3OP3S	100
226.950	C5H6O4S3	100
229.084	C8H21OPS2	71
231.082	C7H19O4PS	56
417.138	C16H37P5S	78
420.882	C7H19P3S7	89
429.154	C17H35O4P3S	82
536.607	C22H95OS5	77
634.871	C12H11O22PS3	93
164.928	H4O2S4	50
393.186	C18H33O5PS	73
377.210	C19H37OPS2	79
429.155	C24H28O3S2	84
522.595	C12H89O16S	77
135.120	C7H18S	100
267.126	C11H22O5S	53
355.230	C20H34O3S	75
623.377	C39H58S3	87
217.081	C10H17OPS	83
207.121	C13H18S	67
220.880	C2H5PS5	100
359.244	C20H38OS2	63
	Average	78
